# Impact of Socioeconomic Disparities on Care and Outcomes of Cancer Patients Presenting With STEMI Between 2005 and 2019; a Nationwide British Study

**DOI:** 10.1002/clc.70135

**Published:** 2025-04-24

**Authors:** Mohamed Dafaalla, Nicholas Weight, Muhammad Rashid, James Nolan, Mamas A. Mamas

**Affiliations:** ^1^ Keele Cardiovascular Research Group, Centre for Prognosis Research, Institute for Primary Care and Health Sciences Keele University Keele UK; ^2^ National Institute for Health and Care Research (NIHR) Birmingham Biomedical Research Centre Birmingham UK

**Keywords:** cancer, outcomes, socioeconomic status, STEMI

## Abstract

**Background:**

While current evidence suggests that the clinical outcomes of STEMI are worse among patients with cancer, it is unknown what role the patient's socioeconomic status plays.

**Methods:**

A nationally linked cohort of STEMI patients (January 2005 to March 2019) was obtained from the MINAP and UK national Hospital Episode Statistics (HES APC) registries. Socioeconomic status was measured using Index of Multiple Deprivation (IMD) score divided into 5 quintiles (quintile 1: most affluent, quintile 5: most deprived). The impact of socioeconomic status on clinical outcomes was assessed using Cox‐proportional‐hazard and competing risk models.

**Results:**

A total of 8459 STEMI admissions with cancer were identified between 01/01/2005 and 30/03/2019 with 1577 patients (18%) from the most deprived quintile. Patients from the most deprived quintile were more frequently female (quintile 5; 29% vs. quintile 1; 24%) and from ethnic minorities (quintile 5; 8% vs. quintile 1; 2%). They were less likely to receive PCI between 2005 and 2009. By 2018 PCI use was higher in quintile 5 (quintile 5; 84% vs. quintile 1;81%). The odds of death at1year was not higher in the most deprived patients. The risk of death was significantly higher at 5 years post‐discharge (HR 1.27, 95% CI 1.10−1.47).

**Conclusions:**

The socioeconomic status impact on outcomes of STEMI patients with cancer can be minimized by delivering equitable inpatient care, particularly PCI. While patients from the most deprived areas have similar odds of survival in the short‐term, they have lower probability of survival in the long‐term.

## Graphical abstract

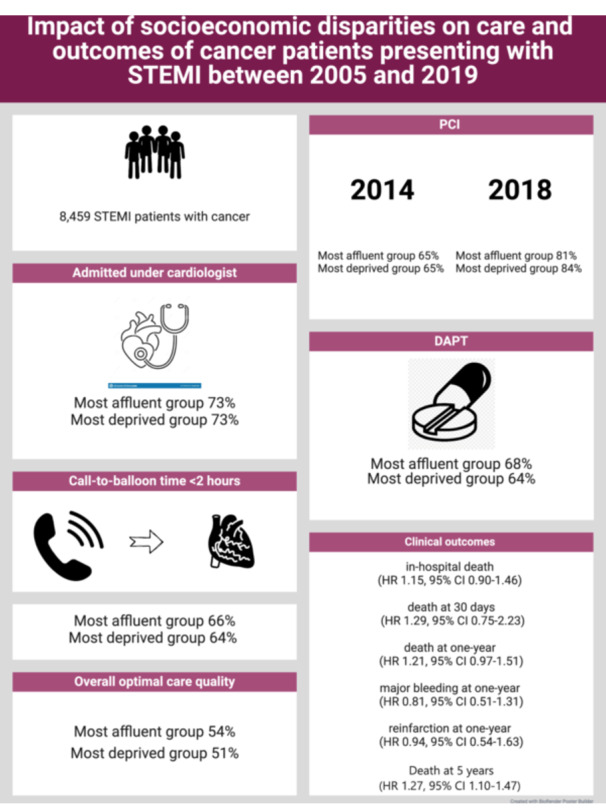

## 1 Introduction

Cardiovascular diseases (CVDs) and cancer are the leading causes of mortality in high‐income countries [1]. The number of cancer survivors has increased over the last two decades as a result of the remarkable improvements in cancer screening and treatment [2]. As a result of this, the number of cancer survivors is expected to increase from 18 million in 2022 to 26 million in 2040 [3]. Cancer patients are now expected to have longer survival which means many of them will develop CVDs. Recent evidence suggest that CVDs are becoming the main cause of death in cancer patients rather than the primary cancer itself [4, 5], with acute myocardial infarction (AMI) being the most common cause of CV mortality [6].

Socioeconomic disparities in AMI presentation, care and outcomes are widely reported. AMI patients living in deprived areas are less likely to receive invasive coronary revascularization, have a prolonged hospital stay, and a lower chance of survival [7, 8]. Whether these disparities are present in cancer patients is still unknown. The UK collects and pools data from 37 indicators of deprivation into a single indicator called Index of Multiple Deprivation (IMD) score which is used to generate detailed reports about the geographical distribution of the deprivation [9]. We used this information in conjunction with data from the national UK Myocardial Infarction National Audit Project (MINAP) heart attack registry and UK national Hospital Episode Statistics Admitted Patient Care (HES APC) registries to investigate the socioeconomic disparities in inpatient care and its impact on the long‐term clinical outcomes in cancer patients following admission with a ST‐elevation myocardial infarction (STEMI).

## 2 Methods

### 2.1 Study Design

This is a population‐based cohort study of cancer patients who were admitted with STEMI in England and Wales between January 2005 and March 2019. Data was obtained via linkages between the MINAP Registry, hospital admission data from the Hospital Episode Statistics (HES) registry, and the National Deaths Registry from the Office for National Statistics (ONS) [10, 11, 12].

MINAP is a national AMI registry which routinely gathers information on the inpatient care of patients hospitalized with AMI in England, Wales, and Northern Ireland. The MINAP data is frequently used to audit the quality of care and for academic research [13, 14, 15]. The database contains information about admission time, patient demographics, comorbidities, clinical characteristics, relevant investigations, inpatient pharmacological and interventional treatments, in‐hospital outcomes, and discharge treatments [16, 17, 18]. The HES APC Registry is a national registry that collects data of all admissions to National Health Service (NHS) hospitals in England [11]. The ONS is the largest independent producer of official statistics in the UK at the national and local levels [12], and it records details of all certified deaths in England and Wales notified in the Civil Registration Deaths Data of the ONS [19]. We used the ONS database to obtain information about the date of death as stated on the medical certificate of cause of death. The databases were linked using a unique identifier for each patient (NHS identifier).

We used the composite opportunity‐based quality indicator (OBQI) to measure the overall care quality [20]. The OBQI is a validated indicator developed by the European Society of Cardiology to facilitate assessment of care quality in patients admitted with AMI [20]. The OBQI reflects the number of care opportunities fulfilled in each hospital (numerator) divided by the number of care opportunities to provide care (denominator) [21]. The score consisted of six evidence‐based care processes: the prescription of aspirin, thienopyridine inhibitor, β‐blocker, angiotensin converting enzyme inhibitor (ACEI/ARB), HMG CoA reductase enzyme inhibitor (statin), and enrollment into a cardiac rehabilitation program at discharge [21]. STEMI patients were then divided into three cohorts based on the OBQI: optimum care (OBQI = 100), intermediate care (OBQI 75−99), and low care quality (OBQI < 75).

### 2.2 Study Population

We identified all STEMI patients with cancer from the MINAP database linked with HES ‐APC. Patients with a diagnosis of cancer were identified from the HES‐APC database by using the International Classification of Diseases (ICD‐10‐CM). Population‐based studies from the national British registries showed that HES database is a reliable source of information on the diagnosis of cancer and AMI diagnosis [22, 23]. The ICD‐10 codes used to describe cancer types in this study are listed in Supporting Information S1: Table 1.

### 2.3 IMD

IMD is a measure of relative deprivation for small areas used to rank neighborhoods across the UK according the output area in which their postcode was located [24]. It is calculated based on 37 separate indicators reflecting seven domains of deprivation including Income score, Employment score, Health score, Education score, Housing score, Crime score, and Living environment score [24, 25]. The IMD score is then divided into five quintiles with quintile 1 representing patients from the most affluent areas and quintile 5 representing patients from the most deprived areas.

### 2.4 Ethical Approval

The study underwent formal ethical approval for the data linkages of MINAP, HES, and ONS registries. Ethical approval was provided by the Health and Care Research Wales (HCRW) and the Health Research Authority (HRA) (REC reference 20/WA/0312) [26].

### 2.5 Clinical Outcomes

The primary clinical outcomes were all‐cause in‐hospital death, death at 30 days, death at 1‐year, major bleeding at 1‐year, and reinfarction at 1‐year post‐discharge.

### 2.6 Statistical Analysis

Continuous variables were expressed as mean and standard deviation (SD) if normally distributed or as median and interquartile range (IQR) if they were skewed. Categorical variables were expressed using percentages. The chi‐square test and *t*‐test were used to test for statistical significance in categorical and continuous variables, respectively. The Kruskal‐Wallis test was used for skewed data. We used the Multiple Imputations with Chained Equations (MICE) algorithm to impute missing data (Supporting Information S1: Table 3). It was assumed that the missing data were missing at random, and 10 imputed data sets were generated and the subsequent analyses were performed on the imputed data set [13, 27, 28]. We used multivariable Cox proportional hazard models to assess the association between socioeconomic and the risk of death post‐discharge. We used competing risk models to assess the association between socioeconomic status and the risk of major bleeding and reinfarction. The variables we adjusted for in these models were age, socioeconomic status, ethnicity, cardiac arrest, cardiogenic shock, left ventricular (LV) ejection fraction, history of angina, previous MI, heart failure (HF), diabetes (DM), hypertension, hypercholesterolemia, peripheral vascular disease, stroke, family history (FH) of CAD, smoking, chronic kidney disease, asthma/COPD, and previous PCI or CABG, coronary revascularization and care quality. Model estimates were reported as hazard ratios (HR) and 95% confidence intervals (95% CI).

## 3 Results

### 3.1 Patients' Characteristics

A total of 8459 STEMI indexed admissions with cancer were identified between January 1, 2005 and March 30, 2019 with 1577 patients (18%) from the most deprived quintile. Patients from the most deprived quintile were younger with lower median age (years) (quintile 5; 72 vs. quintile 1; 76), more frequently female (quintile 5; 29% vs. quintile 1; 24%), or from ethnic minorities (quintile 5; 8% vs. quintile 1; 2%), and were more likely to be smokers (Table 1). Patients from the most deprived quintile had higher burden of cardiovascular comorbidities such as previous AMI (quintile 5; 19% vs. quintile 1;17%), DM (quintile 5; 19% vs. quintile 1; 15%), PVD (quintile 5; 6% vs. quintile 1; 4%), stroke (quintile 5; 11% vs. quintile 1; 6%), and obstructive lung disease (quintile 5; 21% vs. quintile 1; 12%) (Table 2). Supporting Information S1: Table 2 shows the number of patients according to cancer type.

**Table 1:** Patients characteristics.

	Quintile 1 (most affluent)	Quintile2	Quintile 3	Quintile 4	Quintile 5 (most deprived)	*p* value
*N*	**1654**	**1744**	**1806**	**1678**	**1577**	
Age at admission, median (IQR)	76.1 (68.4, 82.7)	76.8 (69.6, 82.9)	75.4 (68.0, 82.1)	74.1 (67.0, 81.5)	72.6 (63.6, 80.1)	< 0.001
Women	397 (24.0%)	422 (24.2%)	446 (24.7%)	466 (27.8%)	462 (29.3%)	< 0.001
Ethnicity						
White	1443 (98.0%)	1510 (97.5%)	1593 (96.7%)	1427 (94.6%)	1332 (91.7%)	< 0.001
BAME	29 (2.0%)	38 (2.5%)	55 (3.3%)	81 (5.4%)	120 (8.3%)	
BMI, median (IQR)	25.6 (22.9, 28.3)	25.4 (22.5, 28.4)	25.7 (22.8, 28.7)	25.4 (22.9, 28.7)	25.6 (22.6, 29.8)	0.55
Cardiac arrest	198 (12.3%)	251 (14.7%)	234 (13.4%)	216 (13.2%)	189 (12.5%)	0.26
Killip class						
Killip class I	641 (70.1%)	605 (67.8%)	586 (64.7%)	561 (66.6%)	530 (67.1%)	0.27
Killip class II	103 (11.3%)	101 (11.3%)	123 (13.6%)	120 (14.3%)	102 (12.9%)	
Killip class III	48 (5.2%)	63 (7.1%)	54 (6.0%)	49 (5.8%)	38 (4.8%)	
Killip class IV	123 (13.4%)	123 (13.8%)	143 (15.8%)	112 (13.3%)	120 (15.2%)	
Left ventricular ejection fraction						
Good	326 (48.5%)	332 (49.6%)	335 (48.0%)	291 (46.2%)	265 (45.5%)	0.58
Moderate	346 (51.5%)	338 (50.4%)	363 (52.0%)	339 (53.8%)	318 (54.5%)	
*Comorbidities:‐*						
PMH of angina	267 (17.8%)	294 (18.6%)	280 (17.7%)	274 (18.7%)	292 (21.3%)	0.098
	259 (17.1%)	282 (17.8%)	312 (19.2%)	307 (20.6%)	264 (19.2%)	0.13
DM	238 (15.1%)	262 (15.8%)	312 (18.4%)	323 (20.3%)	293 (19.6%)	< 0.001
Hypertension	701 (46.6%)	730 (46.4%)	758 (47.2%)	698 (47.1%)	664 (47.6%)	0.97
Hypercholestrolaemia	379 (25.9%)	411 (26.6%)	388 (24.9%)	396 (27.5%)	379 (27.9%)	0.34
Peripheral vascular disease	57 (3.9%)	60 (3.9%)	64 (4.1%)	57 (3.9%)	77 (5.7%)	0.077
Stroke/TIA	90 (6.1%)	111 (7.1%)	130 (8.2%)	130 (9.0%)	144 (10.6%)	< 0.001
FH of CAD	240 (19.3%)	269 (21.3%)	246 (19.8%)	213 (19.3%)	231 (21.3%)	0.53
Smoking status						
Never smoked	661 (45.4%)	622 (40.4%)	611 (38.4%)	533 (36.2%)	428 (30.4%)	< 0.001
Ex‐smoker	612 (42.0%)	672 (43.6%)	677 (42.6%)	580 (39.3%)	528 (37.6%)	
Current smoker	183 (12.6%)	246 (16.0%)	302 (19.0%)	361 (24.5%)	450 (32.0%)	
Chronic kidney disease	95 (6.4%)	114 (7.3%)	105 (6.7%)	87 (6.0%)	71 (5.2%)	0.23
Asthma/COPD	179 (12.1%)	244 (15.7%)	238 (15.1%)	247 (17.0%)	284 (20.9%)	< 0.001
Previous PCI	125 (8.3%)	132 (8.4%)	126 (7.9%)	144 (9.9%)	130 (9.5%)	0.27
Previous CABG	59 (3.9%)	71 (4.5%)	78 (4.9%)	58 (4.0%)	48 (3.5%)	0.34

**Table 2:** Inpatient management.

	Quintile 1 (most affluent)	Quintile2	Quintile 3	Quintile 4	Quintile 5 (most deprived)	*p* value
Call to ballon time < 2 h	920 (66.4%)	937 (64.7%)	962 (64.1%)	828 (61.2%)	825 (64.1%)	0.08
Door to balloon time < 60 min	654 (70.4%)	661 (70.0%)	698 (70.0%)	585 (68.5%)	530 (66.6%)	0.41
Admitted by a cardiologist	1177 (73.2%)	1185 (70.1%)	1193 (68.1%)	1104 (68.3%)	1108 (72.8%)	0.001
Clopidogrel	1234 (78.8%)	1306 (80.5%)	1342 (80.9%)	1259 (81.3%)	1131 (77.8%)	0.078
Glycoprotein IIb/IIIa inhibitors	164 (11.7%)	151 (10.4%)	145 (9.9%)	129 (9.7%)	134 (11.1%)	0.42
Beta blockers	1061 (88.0%)	1082 (88.8%)	1138 (89.1%)	985 (88.3%)	948 (88.8%)	0.91
Warfarin	45 (3.3%)	57 (4.0%)	54 (3.8%)	52 (4.0%)	44 (3.7%)	0.84
Loop diuretics	398 (28.7%)	419 (29.3%)	418 (29.5%)	388 (29.8%)	324 (27.6%)	0.75
Aldosterone antagonists	152 (13.7%)	137 (12.7%)	140 (12.7%)	119 (11.9%)	93 (10.3%)	0.23
Coronary angiogram	903 (63.9%)	857 (59.1%)	921 (61.1%)	782 (57.5%)	718 (58.4%)	0.005
PCI	839 (59.7%)	783 (54.6%)	847 (56.8%)	720 (53.3%)	671 (55.1%)	0.009
CABG	22 (1.5%)	25 (1.6%)	13 (0.8%)	17 (1.2%)	9 (0.7%)	0.070
Coronary revascularization	856 (60.9%)	804 (56.1%)	859 (57.7%)	736 (54.5%)	678 (55.7%)	0.009
*Discharge medications: ‐*						
DAPT	860 (68.4%)	842 (66.2%)	886 (66.4%)	777 (64.5%)	730 (64.2%)	0.19
High‐intensity statin on discharge	1125 (88.0%)	1112 (86.1%)	1189 (87.5%)	1055 (86.7%)	996 (86.8%)	0.65
ACE inhibitor or ARB on discharge for those with moderate and severe LVSD (%)	256 (47.8%)	234 (46.2%)	245 (45.0%)	235 (45.7%)	217 (46.6%)	0.93
Beta‐blocker on discharge for those with moderate and severe LVSD (%)	277 (57.9%)	255 (55.8%)	260 (55.1%)	242 (52.0%)	235 (54.4%)	0.48
Cardiac rehabilitation on discharge	1089 (72.8%)	1113 (71.3%)	1127 (69.6%)	1023 (68.1%)	978 (69.3%)	0.044
*In‐hospital outcomes:‐*						
In‐hospital death	269 (16.3%)	296 (17.0%)	304 (16.8%)	325 (19.4%)	289 (18.3%)	0.11
Bleeding complications	29 (1.8%)	20 (1.2%)	37 (2.1%)	25 (1.5%)	35 (2.3%)	0.12
Reinfarction	35 (2.3%)	39 (2.5%)	51 (3.2%)	38 (2.5%)	26 (1.9%)	0.25

### 3.2 Quality of Care

**Figure 1:**
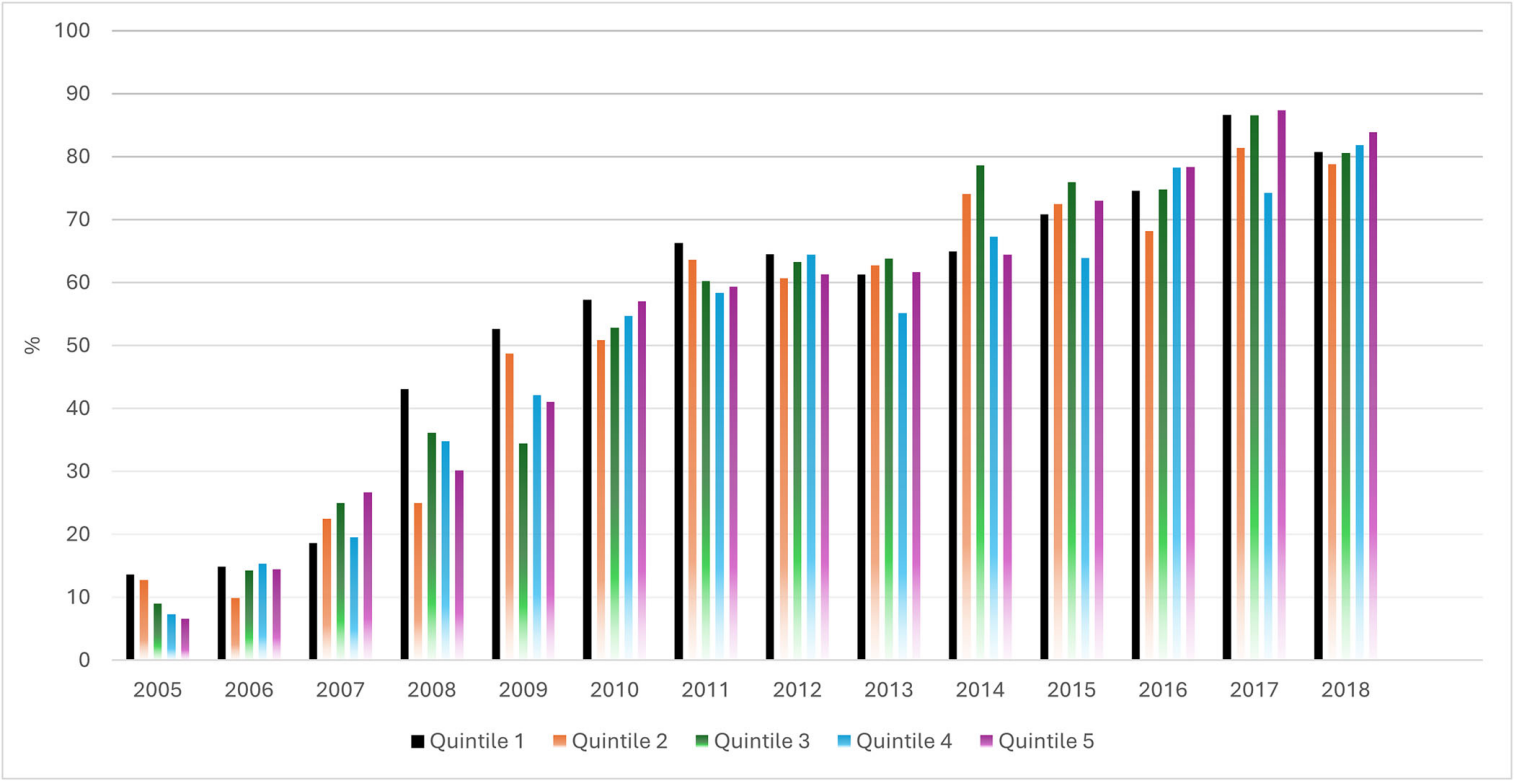
Temporal trends of PCI use and IMD quintiles.

Achievement of Call‐to‐ballon time < 2 h (quintile 5; 64% vs. quintile 1;66%) and door‐to‐balloon time of less than 60 min (quintile 5; 67% vs. quintile 1;70%) in patients from the most deprived areas were comparable to quintile 1 (Table 2). The rate of PCI use increased steadily for all quintiles during the study period. Patients from the most deprived quintile were less likely to receive PCI between 2005 and 2009. By 2014 the gap in PCI use between quintile 5 and quintile 1 was closed (quintile 5; 65% vs. quintile 1;65%). In 2018 the rate of PCI use was higher in the most deprived patients (quintile 5; 84% vs. quintile 1;81%). (Figure 1). A shallow gap was noted in use of DAPT (quintile 5; 64% vs. quintile 1;68%) and cardiac rehabilitation services (quintile 5; 69% vs. quintile 1;73%) (Table 2). During the inpatient stay, patients from the most deprived quintile were likely to receive overall optimal quality of care relatively similar to patients from the most affluent quintile (quintile 5; 51% vs. quintile 1; 54%) (Supporting Information S2: Figure 1).

### 3.3 Clinical Outcomes

**Figure 2:**
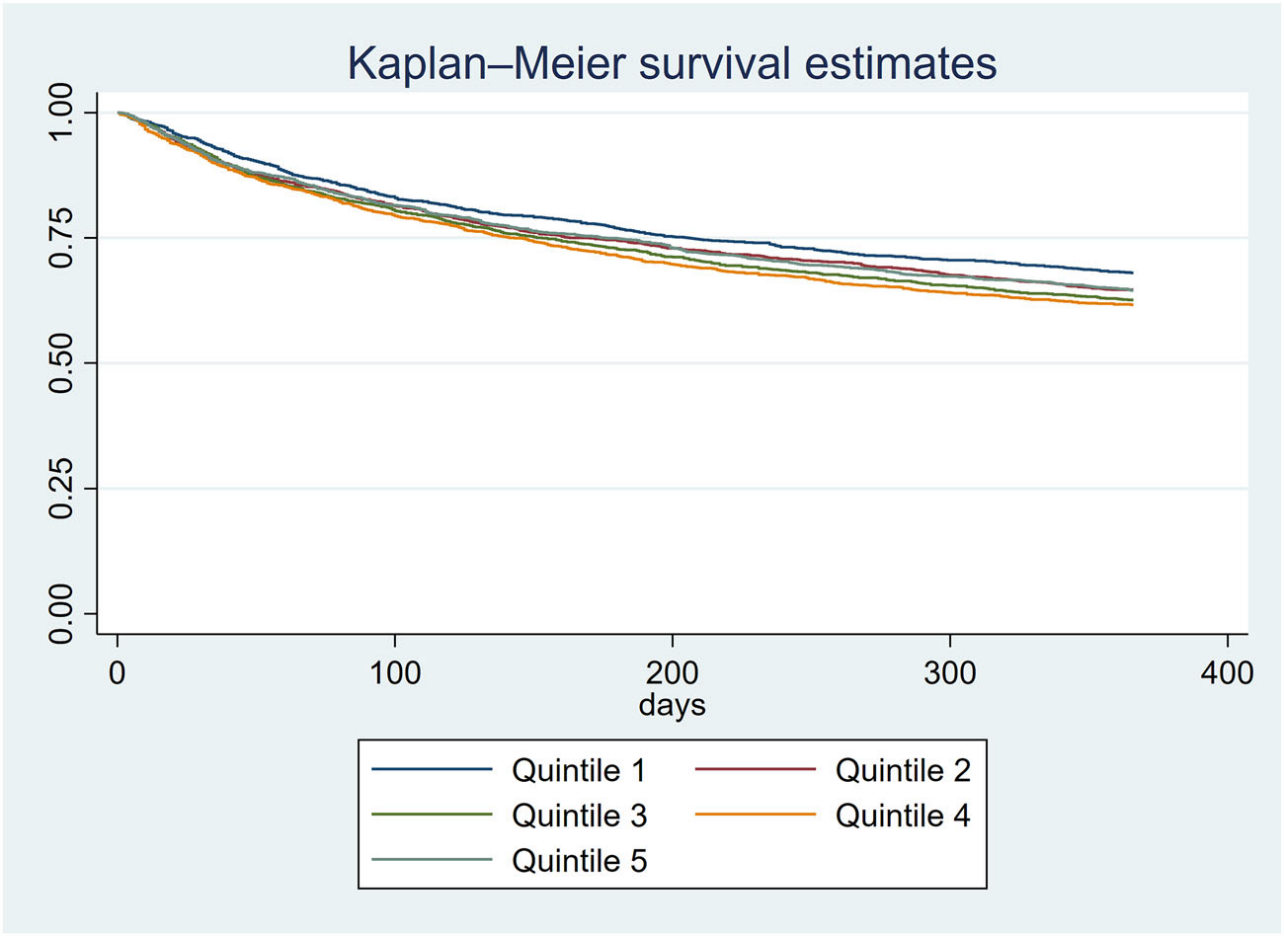
1 year Kaplan−Meier‐survival curve.

After adjusting for patients characteristics, comorbidities, and quality of care the odds of in‐hospital death (HR 1.15, 95% CI 0.90−1.46), death at 30 days (HR 1.29, 95% CI 0.75−2.23), death at 1‐year (HR 1.21, 95% CI 0.97−1.51), major bleeding at 1‐year (SHR 0.81, 95% CI 0.51−1.31), and reinfarction at 1‐year (SHR 0.94, 95% CI 0.54−1.63) were not significantly higher than quintile 1 as shown in Table 3. Figure 2 shows the adjusted 1 year Kaplan−Meier‐survival curve based on the IMD.

**Table 3:** Clinical outcomes.

	Quintile 1 (most affluent)	Quintile2	Quintile 3	Quintile 4	Quintile 5 (most deprived)
In‐hospital death (OR, 95% CI)	Reference	0.89 (0.70−1.13)	1.02 (0.81−1.31)	1.23 (0.97−1.56)	1.1 5 (0.90−1.46)
Death at 30 days (HR, 95% CI)	Reference	1.36 (0.83−2.22)	1.18 (0.71−1.97)	1.39 (0.84−2.34)	1.29 (0.75−2.23)
Death at 1 year (HR, 95% CI)	Reference	1.08 (0.88−1.33)	1.10 (0.89−1.36)	1.15 (0.93−1.43)	1.21 (0.97−1.51)
Bleeding at 1 year (HR, 95% CI)	Reference	1.07 (0.72−1.60)	0.78 (0.50−1.21)	0.86 (0.56−1.34)	0.81 (0.51−1.31)
Reinfarction at 1 year (HR, 95% CI)	Reference	1.02 (0.63−1.67)	0.89 (0.53−1.48)	1.35 (0.83−2.19)	0.94 (0.54−1.63)
*Death beyond the 1 st year after discharge:*					
Death by 2nd year (HR, 95% CI)	Reference	1.06 (0.89−1.28)	1.18 (0.99−1.42)	1.26 (1.04−1.51)	1.21 (1.0−1.47)
Death by 3rd year (HR, 95% CI)	Reference	1.05 (0.89−1.23)	1.15 (0.98−1.35)	1.20 (1.02−1.41)	1.29 (1.09−1.52)
Death by 4th year (HR, 95% CI)	Reference	1.01 (0.87−1.16)	1.07 (0.93−1.24)	1.13 (0.98−1.31)	1.25 (1.08‐1.46)
Death by 5th year (HR, 95% CI)	Reference	1.01 (0.88−1.16)	1.09 (0.96−1.25)	1.14 (1.00−1.31)	1.27 (1.10‐1.47)
*Bleeding beyond the 1 st year after discharge:*					
Bleeding by 2nd year (HR, 95% CI)	Reference	0.92 (0.63−1.34)	0.96 (0.67−1.39)	0.88 (0.62−1.26)	0.98 (0.69−1.39)
Bleeding by 3rd year (HR, 95% CI)	Reference	0.87 (0.61−1.23)	0.83 (0.59−1.18)	0.81 (0.58−1.13)	0.9 1 (0.65−1.26)
Bleeding by 4th year (HR, 95% CI)	Reference	0.87 (0.6−1.24)	0.94 (0.66−1.32)	0.86 (0.61−1.2)	1 (0.72−1.39)
Bleeding by 5th year (HR, 95% CI)	Reference	0.78 (0.55−1.12)	0.9 (0.65−1.26)	0.85 (0.61−1.18)	0.88 (0.64−1.22)
*Reinfarction beyond the 1 st year after discharge:*					
Reinfarction by 2nd year (HR, 95% CI)	Reference	0.90 (0.59−1.38)	0.92 (0.60−1.41)	1.50 (0.99−2.22)	1.01 (0.65−1.59)
Reinfarction by 3rd year (HR, 95% CI)	Reference	0.92 (0.6−1.42)	1.36 (0.93−2)	0.9 (0.6−1.34)	0.79 (0.52−1.2)
Reinfarction by 4th year (HR, 95% CI)	Reference	0.91 (0.59−1.4)	1.37 (0.94−2.01)	0.86 (0.57−1.3)	0.85 (0.57−1.28)
Reinfarction by 5th year (HR, 95% CI)	Reference	0.87 (0.56−1.34)	1.41 (0.97−2.03)	0.86 (0.58−1.29)	0.86 (0.58−1.28)

Supporting Information S2: Figure 2 shows the independent predictors of death at 1 year. The main predictors were suboptimal inpatient care (HR 2.1, 95% CI 1.8−2.4), stroke (HR 1.3, 95% CI 1.0−1.6), obstructive lung disease (HR 1.3, 95% CI 1.3, 95% CI 1.1−1.5), HF (HR 1.5, 95% CI 1.1−2.1), obstructive lung disease (HR 1.3, 95% CI 1.1−1.5), female sex (HR 1.2, 95% CI 1.1−1.4) and coronary revascularization (HR 0.7, 95% CI 0.6−0.8).

### 3.4 Subgroup Analysis‐BAME Ethnicity

The majority of patients from BAME ethnicity belong to the most deprived quintile (quintile 5; 70.7 vs. quintile 1; 74.7%), Patients from the most deprived quintile were younger with lower median age (quintile 5; 20.0% vs. quintile 1;7.4%), and less likely to be women (quintile 5; 15.0% vs. quintile 1; 20.7%). BAME patients from the most deprived quintile had higher burden of cardiovascular comorbidities such as previous AMI (quintile 5; 20.0% vs. quintile 1;7.4%), DM (quintile 5; 32% vs. quintile 1; 28%), stroke (quintile 5; 10.4% vs. quintile 1; 0%), and obstructive lung disease (quintile 5; 14.3% vs. quintile 1; 7.4%). Supporting Information S1: Table 4 shows the characteristics of patients from BAME ethnicity.

We did a sensitivity analysis of death at 1 year based on ethnicity. Patients from the most deprived quintile from both white (HR 1.20, 95% CI 0.69−2.06) and BAME (HR 1.02, 95% CI 0.81−1.28) ethnicities did not have higher risk of death at 1 year compared to quintile 1 (Supporting Information S1: Table 5).

### 3.5 Outcomes Beyond the 1st Year Post‐Discharge

The risk of death among the most deprived quintile was significantly higher by the 2nd year (HR 1.21, 95% CI 1.0−1.47), 3rd year (HR 1.29, 95% CI 1.09−1.52), 4th year (HR 1.25, 95% CI 1.08−1.46), and the fifth year (HR 1.27, 95% CI 1.10−1.47). The risk of bleeding and reinfarction after the 1st year were comparable. Table 3 show the clinical outcomes beyond the 1st year post‐discharge.

## 4 Discussion

Our nationwide cohort study investigating the impact of socioeconomic disparities on management and outcomes of STEMI patients with cancer revealed important findings. Patients from the most deprived areas in the UK are typically younger with a higher proportion of females, more frequently from an ethnic minority background, and have worse cardiometabolic risk factor profiles. A small gap was noted in use of DAPT and cardiac rehabilitation service between the most deprived and most affluent patients. During the inpatient stay, most deprived patients were likely to receive overall optimal quality of care relatively similar to the most affluent patients. The rate of PCI use increased steadily for all quintiles during the study period. Patients from the most deprived quintile were less likely to receive PCI between 2005 and 2009. By 2014 the gap in PCI use between quintile 5 and quintile 1 was narrowed, and in 2018 the rate of PCI use was higher in the most deprived patients. Subsequently, patients from the most deprived areas did not have higher odds of in‐hospital death, death at 30 days, death at 1‐year, major bleeding at 1‐year, or reinfarction at 1‐year after adjusting for patients' characteristics, comorbidities, and quality of care. However, the risk of death after the 1st year was higher in patients from the most deprived areas.

Historic and contemporary evidence recognize the link between the socioeconomic status and clinical outcomes of AMI. For instance, a population‐based study which included all Medicare fee‐for‐service beneficiaries showed that neighborhood socioeconomic disadvantage is associated with higher 30‐day mortality in AMI patients [29]. Data from 6 developed countries indicates that low income patients have elevated admission rates with AMI and lower survival due to the greater burden of CV risk factors, limited opportunities to engage in healthy lifestyle behavior, and obstacles in accessing health care facilities for lower‐income populations [7, 30]. These disparities in management and outcomes of AMI were attributed to differences in health‐related behaviors, comorbid risk factors, access to health care, disparities in inpatient care, and structural racism [31, 32].

While the impact of the socioeconomic status on cancer patients presenting with AMI is not known, the socioeconomic disparities in the cardio‐oncology field are increasingly recognized. For instance, health insurance status, poverty, and geographic region have significant impact on outcomes of treatment‐associated cardiotoxicities [32]. A recent study on cardiovascular mortality of people with and without cancer revealed that although most social disparities narrowed over time, the urban‐rural disparities widened, with greater increase in those with cancer [33]. Furthermore, there is complex interaction between cancer, ethnicity, coronary artery disease and the socioeconomic status. Cancer patients with lower socioeconomic status are frequently from ethnic minorities who are more likely to have higher rates of poverty and lower access to education and health services [8, 34]. Second, coronary artery disease and cancer share common risk factors like obesity, smoking, diabetes mellitus, hypertension, hypercholesterolemia, and renal failure [35]. The burden of these comorbidities is higher in cancer patients with lower socioeconomic status who can present with advanced or severe AMI which can subsequently affect their overall prognosis [35, 36]. The impact of socioeconomic disparities on patients with cancer has not been extensively investigated despite the growing size of this population secondary to improved survival rates over the last two decades as a result of advances in cancer surveillance and management. Moreover, patients with low socioeconomic status and from ethnic minorities are underrepresented in clinical trials which can leave gaps in evidence related to management and risk stratification in AMI patients with cancer [37]. Our study provides a unique opportunity for understanding the impact of socioeconomic status on the clinical outcomes of AMI patients with cancer by clarifying disparities that could be addressed within the health care system and their impact on the clinical outcomes.

This study revealed important differences in the characteristics and inpatient care of STEMI patients with cancer from the most deprived areas in the UK. Patients from the most deprived areas were younger, more frequently females, and more likely to be from ethnic minorities. Patients from the most deprived areas also have greater burden of CV risk factors such as DM, PVD, stroke, and obstructive lung disease probably because of the reduced ability to adopt healthy lifestyle behavior [7, 30]. Our study also demonstrated that compared to the most affluent patients, patients from the most deprived areas had a slightly lower chance of receiving evidence‐based medications like DAPT, beta blockers, and ACE inhibitors. In fact, suboptimal inpatient care and coronary revascularization were the main predictor of patients' survival at 1 year.

While the current evidence shows a strong association between socioeconomic status and clinical outcomes of AMI in general, our study shows that the socioeconomic status has limited impact on the clinical outcomes of STEMI patients with cancer in the 1st year post‐discharge. After adjusting for patients' characteristics and quality of care patients from the most deprived areas did not have increased odds of death, bleeding, or reinfarction which can be attributed to multiple factors. First, patients with cancer have equitable access to UK health care services before and after hospital arrival. Our study shows that the differences in use of call‐to‐balloon time between patients from the most deprived and most affluent areas were shallow probably because of implementation of British National service frameworks. In contrast to the United States, the UK has a publicly funded universal health care system where national frameworks for cardiac disease were implemented to provide access to the best possible treatment in timely fashion for all patients with heart disease, including PCI [38]. Second, disparities in the inpatient care were minimal as well. Although patients from the most deprived areas had a slightly lower chance of receiving evidence‐based medications during their admission, they had similar door‐to‐balloon time, similar access to a cardiologist, and comparable access to PCI particularly after 2012 which is concomitant with the expansion of the PCI services in UK in 2012 as part of the National service frameworks [39]. As a result, patients from the most deprived quintile receive overall optimal quality of care relatively similar to patients from the most affluent quintile. It is also worth noting that the proportion of patients from ethnic minorities is much lower in the UK which is an important difference between the United States and the UK. White ethnicity constitutes around 58% of the United States population compared to around 82% of the UK population [40, 41]. Notably, the risk of death in patients from the most deprived areas is higher after the 1st year post‐discharge. While our data doesn't explain this observation, these findings iterate the importance of long‐term follow up particularly for patients from the most deprived areas. Further research is required to explore the factors that contribute to the higher long‐term risk of death in patients from the most deprived regions.

The United Kingdom established the NHS aiming to provide equitable health care to the whole population. There was growing evidence that patients living in the most deprived areas were disadvantaged by lower revascularisation rates, despite having a higher level of need. Subsequently the national service frameworks for cardiac disease were established as a health service intervention to provide access to the best possible treatment in timely fashion for all with heart disease [42]. As a result, patients diagnosed with STEMI by paramedics are now taken directly to the nearest primary PCI center aiming for a door to ballon time less than 90 min. Previously patients were thrombolysed at their nearest general hospital and then transferred, often days later for PCI. Moreover, higher budget allocation for cardiac services allowed more patients with cardiac disease to be treated, with substantial capital investment in cardiac catheter laboratories. These changes to health policies helped close the gap in care quality provided between the most affluent and most deprived regions [8, 39].

To the best of our knowledge this is the first study that provides an insight on the impact of socioeconomic disparities on care and clinical outcomes of STEMI patients with cancer. However, there are a few limitations to be considered when interpreting the results of this study. The study design is observational which limits our ability to make causality conclusions. The exact staging of the cancer and details of cancer therapy were not available and therefore we didn't manage to explore the interaction between socioeconomic status. cancer progression, and STEMI outcomes. Details of the culprit lesions, and number of vessels treated for patients who had PCI were not available as they were not routinely collected in the databases. The registries don't routinely collect data related to quality of life like quality‐adjusted life years (QALYs) as well.

## 5 Conclusion

To conclude, this nationwide study from the UK shows that the impact of socioeconomic status on the clinical outcomes of STEMI patients with cancer can be minimized by delivering equitable inpatient care. Policy makers and health care leaders in areas with large ethnic minority population or significant socioeconomic disparities are encouraged to implement effective strategies to improve the quality of inpatient care, particularly use of PCI, which will subsequently reduce the impact of the socioeconomic disparities on clinical outcomes of STEMI patients with cancer. While patients from the most deprived areas have similar odds of survival in the short‐term, they have lower probability of survival in the long‐term. Further studies are required to understands the factors behind this observation.

## Conflicts of Interest

The authors declare no conflicts of interest.

## Supporting information

Supporting.

Supporting fig 2.

## Data Availability

The data underlying this article cannot be shared publicly due to [describe why the data cannot be shared, for example, for the privacy of individuals that participated in the study]. The data will be shared on reasonable request to the corresponding author.
