# Supplementary material for: Impact of Socioeconomic Disparities on Care and Outcomes of Cancer Patients Presenting With STEMI Between 2005 and 2019; a Nationwide British Study
Source: Clin Cardiol. 2025 Apr 24;48(4):e70135. doi: 10.1002/clc.70135 (PMC12019704; doi:10.1002/clc.70135)
Supplement: Supplementary file 1 — Supporting. [file CLC-48-e70135-s001.docx]

Supplements

Supplementary table 1: ICD10 codes

| Myocardial infarction | |
| --- | --- |
| I21-I22 | Acute myocardial infarction |
|  | Cancer codes |
| C81-C86, C88, C90-C96 | Haematological cancer |
| C34, C38.4 C38.8 C39.9 | Lung Cancer |
| C61 | Prostate cancer |
| C50 | Breast cancer |
| C18-C20 | Colorectal cancer |
|  | Other gastrointestinal malignancies: - |
| C15 | Oesophagus |
| C16 | Stomach |
| C17 | Small intestine |
| C21 | Anal |
| C22, C23, C24, C25  C26 | Liver, biliary tract, and pancreas  Other digestive organs |
|  | Skin cancer: - |
| C43  C44 | Melanoma  Other skin cancers |
| C64- C68 | Malignancy of the kidneys, ureters, and bladder: - |
|  | Metastatic cancer (Secondary malignant neoplasm): - |
| C77 | of the lymph nodes |
| C78 | of respiratory and digestive organs |
| C79 | of unspecified site |
| C80, C76 | Malignant neoplasm without specification of site |
|  | Malignancy of the oral cavity and pharynx: - |
| C01X, C029, C030, C049  C051, C07X, C099  C10-C14 | Oropharynx/Nasopharynx |
| C32 | Laryngeal cancer |
| C41, C49 | Cancer of the bone and connective tissue |
| C51- C58 | Gynaecologic malignancies |
| C71, C72 | Malignant neoplasm of the brain and central nervous system |

Supplementary table 2: Number of patients according to cancer type

|  | Count |
| --- | --- |
| All cancers | 8,459 |
| Prostate cancer | 2,523 |
| Lung cancer | 1,117 |
| Colon cancer | 669 |
| Haematologic malignancies | 1,254 |
| breast cancer | 388 |

Supplementary table 3: Imputed data

|  | **Complete** | **Imputed** | **Total** |
| --- | --- | --- | --- |
| **Ethnicity** | 7628 | 831 | 8459 |
| **OHCA** | 8206 | 253 | 8459 |
| **Cardiogenic shock** | 4345 | 4114 | 8459 |
| **LV function** | 3253 | 5206 | 8459 |
| **Previous angina** | 7493 | 966 | 8459 |
| **Previous AMI** | 7594 | 865 | 8459 |
| **HF** | 7451 | 1008 | 8459 |
| **DM** | 8013 | 446 | 8459 |
| **HTN** | 7559 | 900 | 8459 |
| **Hypercholesterolemia** | 7366 | 1093 | 8459 |
| **PVD** | 7384 | 1075 | 8459 |
| **Stroke** | 7435 | 1024 | 8459 |
| **FH of CAD** | 5943 | 2516 | 8459 |
| **Smoking** | 7466 | 993 | 8459 |
| **CKD** | 7416 | 1043 | 8459 |
| **Asthma/COPD** | 7414 | 1045 | 8459 |
| **Previous PCI** | 7482 | 977 | 8459 |
| **Previous CABG** | 7490 | 969 | 8459 |
| **DAPT** | 6206 | 2253 | 8459 |

Supplementary table 4: Characteristics of patients from BAME ethnicity

|  | **Quintile 1(most affluent)** | **Quintile2** | **Quintile 3** | **Quintile 4** | **Quintile 5 (most deprived)** | **p-value** |
| --- | --- | --- | --- | --- | --- | --- |
| N | 29 | 38 | 55 | 81 | 120 |  |
| Age at Admision, median (IQR) | 74.7 (70.1, 79.7) | 74.4 (65.4, 80.0) | 73.3 (65.0, 79.4) | 73.5 (62.0, 80.4) | 70.7 (60.9, 78.4) | 0.31 |
| Women | 6 (20.7%) | 7 (18.4%) | 16 (29.1%) | 15 (18.5%) | 18 (15.0%) | 0.30 |
| BMI | 23.9 (22.5, 26.4) | 26.4 (23.7, 29.4) | 22.6 (20.6, 25.4) | 24.1 (22.9, 27.3) | 24.6 (23.1, 29.4) | 0.11 |
| Previous MI | 2 (7.4%) | 6 (16.7%) | 14 (26.9%) | 13 (17.6%) | 21 (20.0%) | 0.31 |
| DM | 8 (28.6%) | 7 (19.4%) | 17 (32.7%) | 30 (39.0%) | 38 (32.8%) | 0.35 |
| Hypertension | 14 (50.0%) | 15 (42.9%) | 33 (63.5%) | 44 (59.5%) | 64 (58.2%) | 0.34 |
| Hypercholestrolaemia | 7 (25.9%) | 10 (29.4%) | 19 (38.8%) | 28 (38.4%) | 38 (35.2%) | 0.72 |
| Peripheral vascular disease | 2 (7.7%) | 1 (2.9%) | 3 (5.9%) | 4 (5.4%) | 2 (1.9%) | 0.57 |
| Stroke/TIA | 0 (0.0%) | 1 (2.9%) | 2 (3.9%) | 2 (2.8%) | 11 (10.4%) | 0.086 |
| FH of CAD | 5 (20%) | 1 (4%) | 12 (26%) | 8 (14%) | 10 (12%) | 0.11 |
| Ex-smoker | 7 (25.0%) | 20 (57.1%) | 18 (34.0%) | 14 (19.4%) | 28 (26.4%) |  |
| Current smoker | 3 (10.7%) | 0 (0.0%) | 8 (15.1%) | 13 (18.1%) | 25 (23.6%) |  |
| CKD | 0 (0.0%) | 2 (5.7%) | 3 (5.9%) | 4 (5.5%) | 5 (4.8%) |  |
| Asthma/COPD | 2 (7.4%) | 6 (17.1%) | 8 (15.1%) | 8 (10.8%) | 15 (14.3%) | 0.75 |
| Previous PCI | 2 (7.4%) | 2 (5.6%) | 8 (15.4%) | 7 (9.6%) | 17 (16.2%) | 0.34 |
| Previous CABG | 1 (3.7%) | 1 (2.8%) | 6 (11.5%) | 4 (5.4%) | 4 (3.8%) | 0.29 |

Supplementary table 5: sensitivity analysis of death at 1 year based on ethnicity.

|  | **Quintile 1**  **(most affluent)** | **Quintile2** | **Quintile 3** | **Quintile 4** | **Quintile 5**  **(most deprived)** |
| --- | --- | --- | --- | --- | --- |
| White ethnicity | reference | 1.35(0.83-2.22) | 1.16(0.68-1.98) | 1.28(0.78-2.16) | 1.20(0.69-2.06) |
| BAME ethnicity | Reference | 1.12(0.91-1.39) | 1.27(1.03-1.57) | 1.18(0.95-1.47) | 1.02(0.81-1.28) |

Supplementary figure 1: Proportion of patients received optimal quality of care (OBQI=100) and IMD quintiles
