# Supplementary figures and images for: Impact of Socioeconomic Disparities on Care and Outcomes of Cancer Patients Presenting With STEMI Between 2005 and 2019; a Nationwide British Study
Source: Clin Cardiol. 2025 Apr 24;48(4):e70135. doi: 10.1002/clc.70135 (PMC12019704; doi:10.1002/clc.70135)

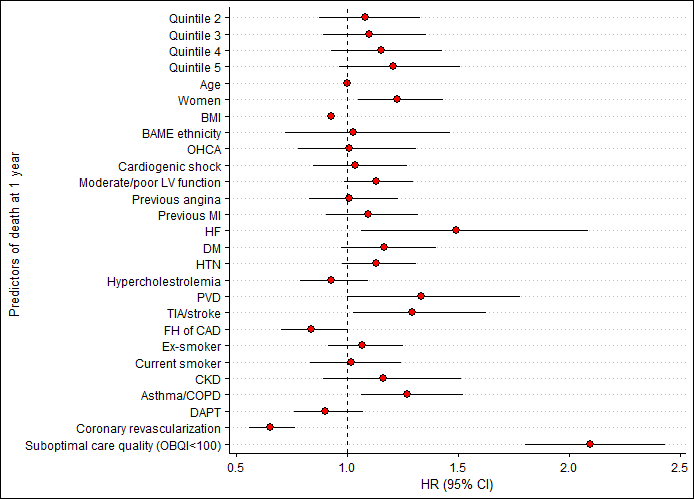

Supplement: Supplementary file 2 — Supporting fig 2. [file CLC-48-e70135-s002.tiff]
